# Supplementary material for: Artificial non-monotonic neurons based on nonvolatile anti-ambipolar transistors
Source: Nat Commun. 2025 Apr 3;16:3188. doi: 10.1038/s41467-025-58541-8 (PMC11968844; doi:10.1038/s41467-025-58541-8)
Supplement: Supplementary file 1 — Supplementary Information [file 41467_2025_58541_MOESM1_ESM.pdf]

Supplementary Information

Artificial non-monotonic neurons based on nonvolatile anti-ambipolar transistor

Yue Pang[1], Yaoqiang Zhou [1, 2,] *, Shirong Qiu[1], Lei Tong[1], Ni Zhao[1], Jian-Bin Xu[1,] *

1, Department of Electronic Engineering and Materials Science and Technology Research Center, The Chinese University of Hong Kong, Hong Kong SAR, China

2, Department of Electronics and Nanoengineering, Aalto University, Espoo 02150, Finland

*Corresponding author: jbxu@ee.cuhk.edu.hk

Supplementary Note. 1 Origin of anti-ambipolar transfer characteristic in the junction transistor

Supplementary Figure 1a shows the schematic of the proposed anti-ambipolar transistor based on n-type MoS_2 and p-type WSe_2 transistors. The equivalent circuit diagram as shown in **Supplementary Figure 1b**. The drain current I_{DS} is determined by the MoS_2 and WSe_2 resistance, R_n and R_p, respectively, connected in series.[1] Hence, I_{DS} is expressed by **Eq. 1**.

$$I_{DS}=\frac{V_{DS}}{R_n+R_p} \tag{1}$$

Generally, I_{DS} is determined by the segment of the circuit with the highest resistance as indicated in **Supplementary Figure 1c**. When a negative gate voltage is applied ($V_G < 0$ V), the gate electric field induces strong electron depletion on the MoS_2 side, resulting in an increase in R_n, which becomes the primary resistor determining I_{DS}. Hence, the left side of the bell-shaped transfer curve is predominantly influenced by the transfer characteristics of the MoS_2 transistor, as shown in **Supplementary Figure 1c**. Conversely, with a positive gate voltage ($V_{GS} > 0$ V), the gate electric field induces strong hole depletion, making R_p the dominant factor in the I_{DS}. Therefore, the right side of the bell-shaped curve is predominantly influenced by the transfer characteristics of the WSe_2 transistor. The alternation of the resistance values of R_n and R_p results in the appearance of a bell-shaped transfer curve peak. Consequently, when the shapes of the transfer curves of n-type MoS_2 and p-type WSe_2 transistors are determined, their relative positions will dictate the position and shape of the bell-shaped transfer curve.

Besides, noted that the anti-ambipolar attributes are realized by utilizing the sub-threshold transistor characteristics. Therefore, to obtain the symmetric anti-ambipolar properties, we need to ensure similar sub-threshold swing (*SS*) values for the complementary transistors. Ideally, the *SS* values for both n-and p

transistors should be 60 mV/dec. By improving the interface quality and reducing the interface defect density, we can make *SS* values of complementary transistors approach the ideal value simultaneously, resulting in a more symmetrical bell-shaped transfer curve.

Supplementary Note. 2 Physiological perspective and implementation of pulse encoder

2.1 The physiological perspective of the pulse encoder

The pulse encoder process serves as a representation of how cardiomyocytes, the muscle cells found in the heart, contribute to the cardiovascular physiological functions of the human body. The contraction strength and rhythm of heart muscle cells in the atria and ventricles play a significant role in the overall function of the cardiovascular system [2] [3]. This process involves the regulation of blood pressure and heart rate, which in turn reflect the force of ventricular contractions responsible for pumping blood from the heart to peripheral organs and tissues [3]. These physiological parameters can be encoded as the amplitude of the pulse input. Additionally, heart rate corresponds to the rhythmic activity of heart muscle cells [4] and can be encoded as the pulse width. The artificial nonmonotonic neuron emulates the interaction of peripheral organs and systems in the human body, akin to how the heart rate corresponds to the rhythmic activity of heart muscle cells. The nonmonotonic response mirrors the cardiovascular system's behaviour, which integrates signals from peripheral organs and reacts to pulse stimulation.

2.2 Introduction of the MIMIC and PWH dataset

The MIMIC dataset is an open-access database provided by Beth Israel Deaconess Medical Center in Boston, United States, which contains real-time clinical recordings from a diverse patient population [1,2]. It contains ECG, ABP (arterial blood pressure), PAP (pulmonary arterial pressure), CVP (central venous pressure), and PLE (fingertip plethysmograph) recorded signals, etc. Most importantly, this well-established dataset offers a sufficiently large sample size, ensuring a broad range of resting heart rate (RHR) and systolic blood pressure (SBP) values, which is critical for training and validating our device. The PWH-Elderly and PWH-Young are two self-built datasets. The PWH datasets were collected from the Prince of Wales Hospital in Hong and were approved by the Joint Chinese University of Hong Kong–New Territories East Cluster Clinical Research Ethics Committee, with consent obtained from the participants.[1] The recruited subjects

will be categorized as PWH-Elderly if they are older than 65 years of age (*n*=38); conversely, subjects younger than 35 years of age will be categorized as PWH-Young (*n*=23). The MIMIC dataset is an open-access database provided by Beth Israel Deaconess Medical Center in Boston, United States, which contains real-time clinical recordings from a diverse patient population,[2,3] and we randomly selected RHR and SBP data from 47 subjects in MIMIC-I as part of the training dataset for our device.

2.3 Implementation of pulse encoder

In the data processing, we extracted the peak values from the arterial waveform from the datasets which corresponding to the value of systolic blood pressure (SBP) in each cardiac cycle. furthermore, within the same record of arterial waveform, we calculated the peak-peak interval to obtain the resting heart rate (RHR). This technique allows accurate and synchronous extraction of both RHR and SBP. The practical observation reveals a non-monotonic relationship between the homeostasis of SBP and RHR [5]. This means that when these parameters deviate from the equilibrium level maintained by homeostasis, it negatively impacts the cardiovascular system. As a solution, a homeostasis pulse encoder strategy was developed for both amplitude and width pulse input encoders.

To align the SBP and RHR with the device response and allow for distinct differentiation, we utilize an exponential coding strategy to compress the SBP range and direct linear mapping for the RHR (as shown in **Figure. 5a**). These encoders are easy to implement and commonly used ,[6, 7] and their combination works effectively with the device characteristics to achieve the feature coding function. Therefore, in the context of pulse input amplitude from a physiological standpoint, both SBP and RHR are involved in the encoding process while the pulse width is manipulated by the RHR. The device has an input voltage range of [0~30] V and a manipulation width of [0.2 -1] ms. Therefore, the pulse width encoder is expressed in **Eq.2**.

$$\text{Pulse\_Width (ms)}=\begin{cases}0.2\cdot\text{round}\left(\frac{4\cdot(\text{RHR-120})}{70}+5\right), \text{RHR}\leq 128\\ 1, \text{RHR}>128\end{cases} \quad (2)$$

On the other hand, the pulse amplitude encoder is divided into three distinct steps.

1) The initial step involves creating the SBP encoder through the transformation of SBP into a power of 2 representation. This process is essential in establishing a unique nonlinear encoder value, which is then utilized for the integration into an encoder table. The SBP encoder formulation (**Eq.3**) is developed by

aligning the values x=[70 105 160], where these values are within the practical SBP range [50, 210] mmHg, with the corresponding SBP encoder powers=[1 3 5],

$$SBP_{encoder_{power}}=0.0437 \cdot SBP+-1.8826,\ 50 \leq SBP \leq 210 \tag{3}$$

2) Secondly, we generate a combination encoder of the SBP encoder and HR encoder as shown in **Eq.4**.

$$Combination\_encoder=2^{SBP_{encoder_{power}}} \cdot Pulse_{Width}/0.2 \tag{4}$$

3) Finally, the encoder value of the combination encoder (CE) will ultimately determine the amplitude of the device input signal. If the CE is less than 12, the pulse amplitude will be lower; for CEs ranging between 12 and 30, the pulse amplitude will be normal; and for CEs exceeding 30, the pulse amplitude will be higher. Therefore, the pulse amplitude encoder is expressed in **Eq. 5**.

$$Pulse\_Amplitude\ (V)=\begin{cases} \frac{8 \cdot (CE-12)}{10}+13, & CE<12 \\ \frac{18 \cdot (CE-30)}{18}+31, & 12 \leq CE<30 \\ \frac{4 \cdot (CE-450)}{420}+35, & 30 \leq CE \end{cases} \tag{5}$$

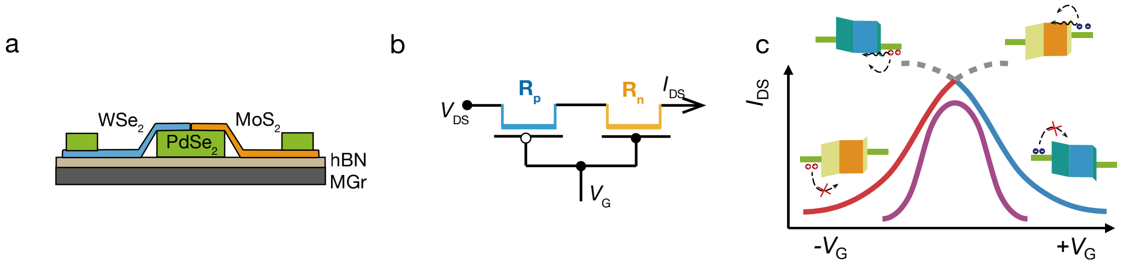

Supplementary Fig. 1 Schematic illustration of the working mechanism of anti-ambipolar transistor. **(a)** The schematic of our proposed anti-ambipolar (AAT) transistor based on n-type MoS_2 and p-type WSe_2 transistors**. (b)** Circuit diagram of AAT device. **(c)** Illustration of the AAT device, and indices to determine the bell-shaped curve properties.

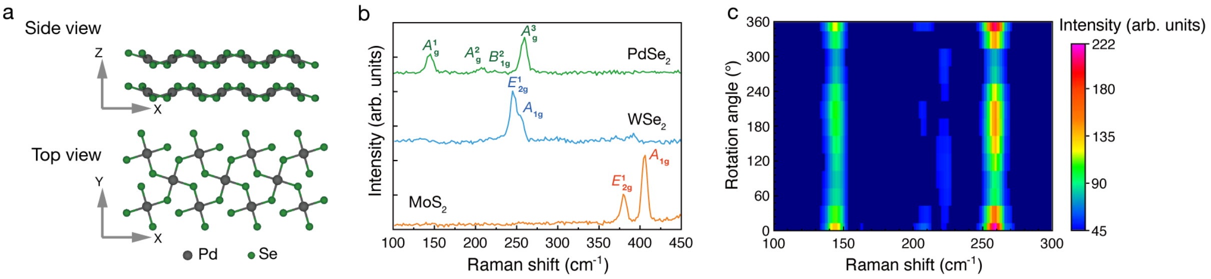

Supplementary Fig. 2 Material characterization of $PdSe_2$. **(a)** The lattice structure of $PdSe_2$ flakes with a puckered pentagonal. The unit cell vectors are denoted by the *x*, *y*, and *z*. Grey and green globes denote the Pd and Se atoms, respectively. **(b)** Raman spectra of mechanically exfoliated $PdSe_2$, WSe_2, and MoS_2 flakes. **(c)** Angle-resolved Raman spectra of exfoliated $PdSe_2$. The intensities of the A_g modes exhibit a 2-lobed shape with four maximum-intensity angles at ~0° and ~180°.

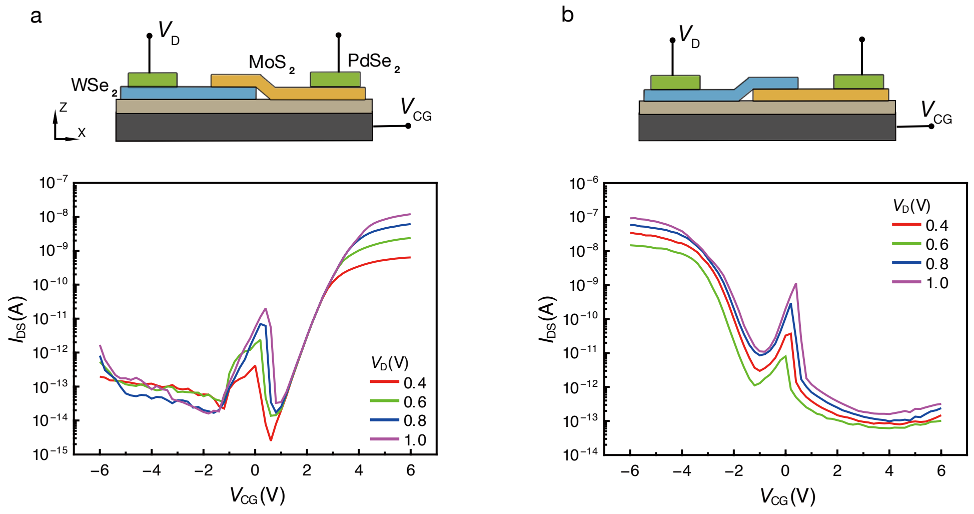

Supplementary Fig. 3 (a) Schematic and transfer curves of WSe_2/MoS_2 contact device (WSe_2 on top). **(b)** Schematic and transfer curves of WSe_2/MoS_2 contact device (MoS_2 on top).

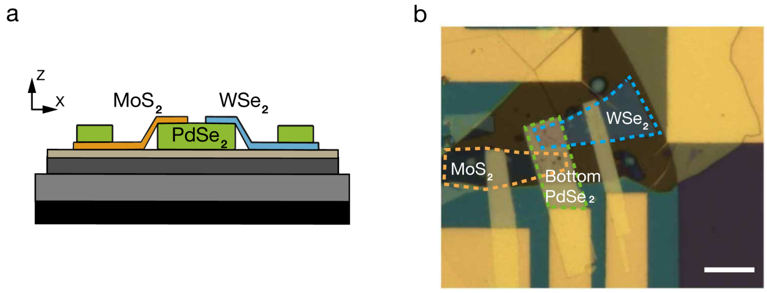

Supplementary Fig. 4 (a) The side view schematic of MoS_2/$PdSe_2$/WSe_2 junction transistor. **(b)** The optical image of MoS_2/$PdSe_2$/WSe_2 junction transistor. The MoS_2 and WSe_2 channels were not connected directly; instead, they are individually contacted with the bottom $PdSe_2$ electrode. Scale bar: 5 µm.

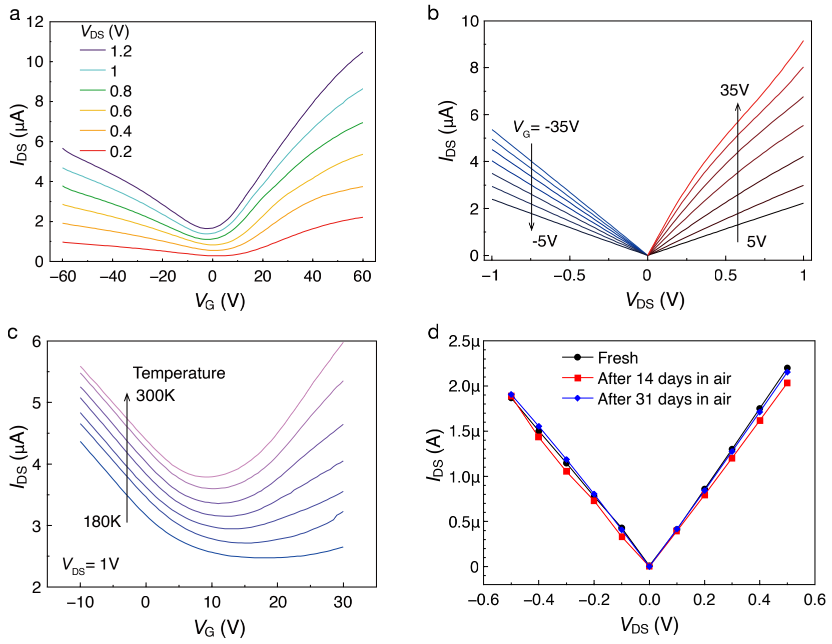

Supplementary Fig. 5 Electrical characterization of $PdSe_2$ transistor. **(a)** Transfer curves of $PdSe_2$ transistor with varying V_{DS} ranging from 0.2 V to 1.2 V. The Au films were dry transferred as contact electrodes. As V_{DS} increases, the transistor gradually exhibits obvious ambipolar transfer characteristics. **(b)** Output curves of $PdSe_2$ transistor at varying V_G. **(c)** Temperature-dependent transfer curves of $PdSe_2$ transistor. The transfer curves convert to unipolar p-type transfer characteristics as temperature decreases. **(d)** Output characteristics of $PdSe_2$ transistor measured during various times in the air. The conduction exhibited negligible degradation after 31 days of exposure to the air.

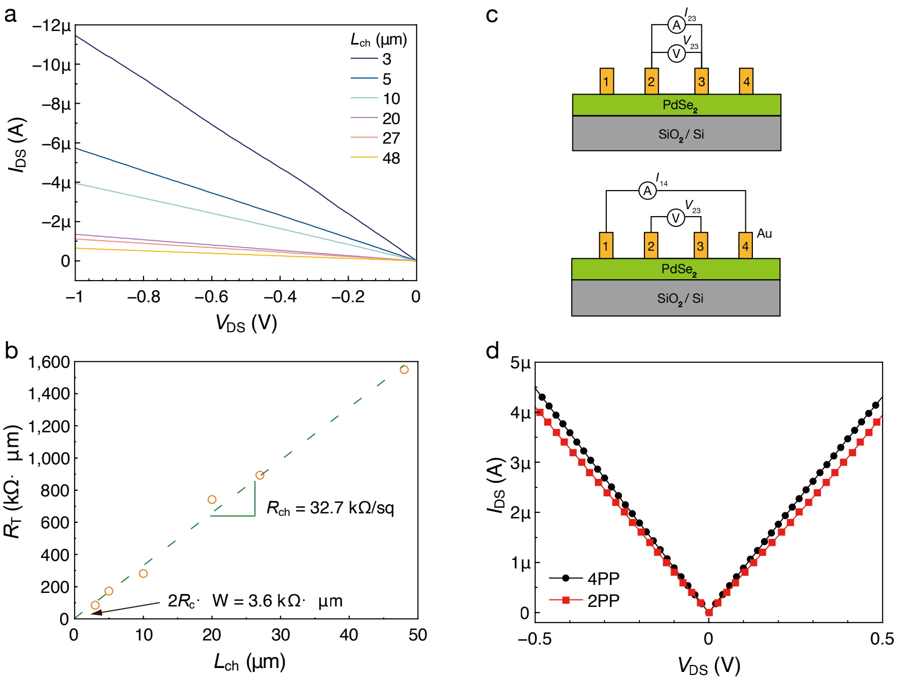

Supplementary Fig. 6 Contact performance of $PdSe_2$ electrode. **(a)** I_{DS}-V_{DS} curves of $PdSe_2$ with different lengths. The linear I_{DS}-V_{DS} relationship indicated the Ohmic contact between $PdSe_2$ and Au electrode. **(b)** TLM contact resistance of Au electrodes on $PdSe_2$. **(c)** Schematic of two-point-probe (2PP) and four-point-probe (4PP) electrical measurement. **(d)** I_{DS}-V_{DS} curves of $PdSe_2$ measured by the 2PP and 4PP method.

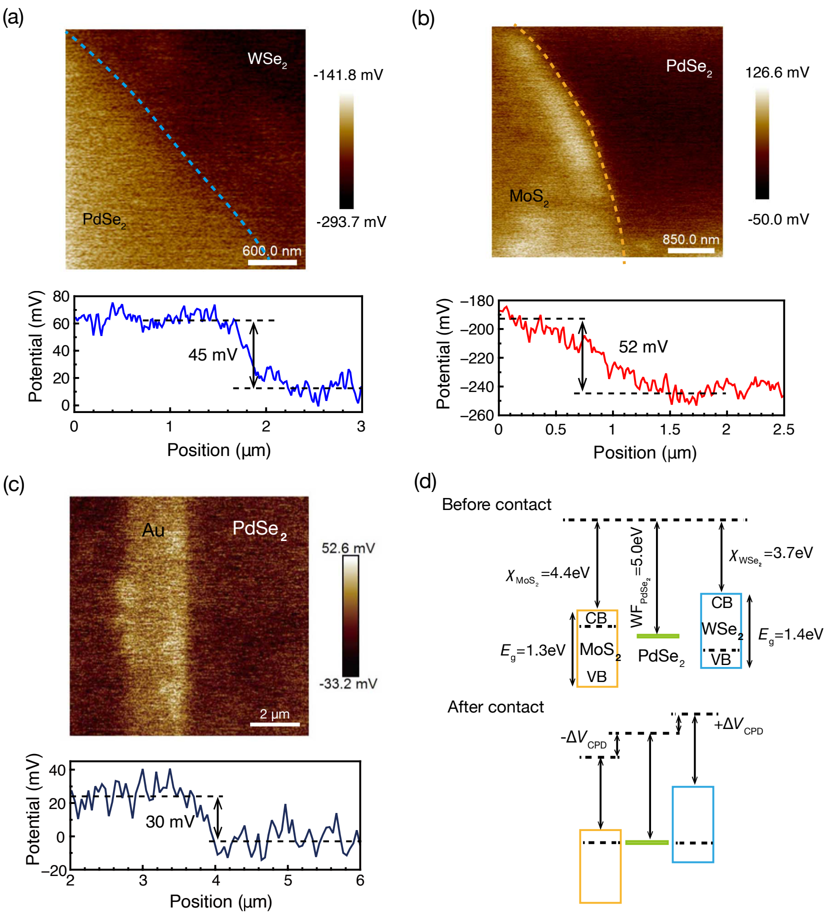

Supplementary Fig. 7 Surface potential difference measured by Kelvin probe force microscopy. **(a)** Potential image of $PdSe_2/WSe_2$ junction and the measured surface potential difference, the Fermi level of WSe_2 is 45 mV lower than the work function of $PdSe_2$. Scale bar: 600 nm. **(b)** Potential image of $PdSe_2/MoS_2$ junction and the measured surface potential difference, the Fermi level of MoS_2 is 52 mV higher than the work function of $PdSe_2$. Scale bar: 850 nm. The opposite surface potential difference between $PdSe_2/WSe_2$ junction and $PdSe_2/MoS_2$ junction indicates the $PdSe_2$ work function aligns near the valence band of WSe_2 and the conduction band of MoS_2. **(c)** Potential image of $PdSe_2$/Au junction and the measured surface potential difference. Scale bar: 2 μm. **(d)** The band alignment diagrams of $PdSe_2$, WSe_2, and MoS_2 before and after contact.

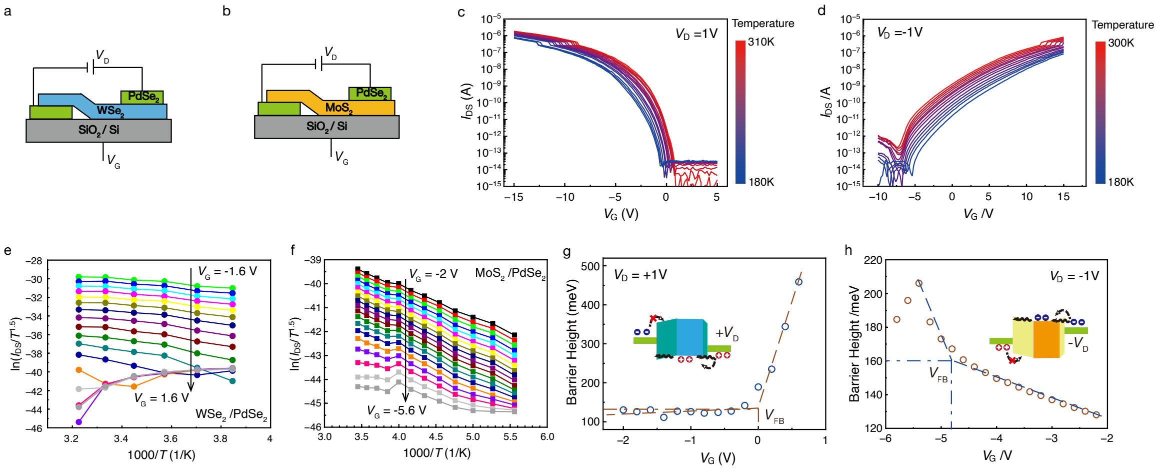

Supplementary Fig. 8 Schottky barrier of $PdSe_2$-contacted WSe_2 and MoS_2 are calculated by 2D thermionic emission equation: $I_{DS} = \left[A^* T^{1.5} \exp\left(-\frac{q\Phi_B}{k_B T}\right)\right]\left[\exp\left(\frac{qV_{DS}}{k_B T}-1\right)\right]$. Where I_{DS} is the saturation current density, A^* is the effective Richardson–Boltzmann constant, T is temperature, q is the electron charge, Φ_B is the Schottky barrier height, and k_B is the Boltzmann constant. The Schottky barrier height is extracted under a flat-band gate voltage condition, which was responsible for the start of deviations from the linear behavior. **(a, b)** Schematic structure of **(a)** $PdSe_2$-WSe_2, **(b)** $PdSe_2$- MoS_2 contact. **(c, d)** I_{DS}-V_G curves of **(c)** $PdSe_2$-WSe_2 transistor **(d)** $PdSe_2$- MoS_2 transistor at varying temperatures ranging from 310 K to 180 K. **(e)** Arrhenius plots of $\ln(I_{DS}/T^{1.5})$ versus 1000/K at varying gate voltages from -1.6 V to +1.6 V of $PdSe_2$-WSe_2 contact. **(f)** Arrhenius plots of $\ln(I_{DS}/T^{1.5})$ versus 1000/K at varying gate voltages from -2 V to -5.6 V of $PdSe_2$/MoS_2 contact. **(g, h)** Barrier heights of the **(g)** $PdSe_2$-WSe_2 and **(h)** $PdSe_2$-MoS_2 Schottky junctions as a function of V_G. The Schottky barrier height is extracted under a flat-band voltage.

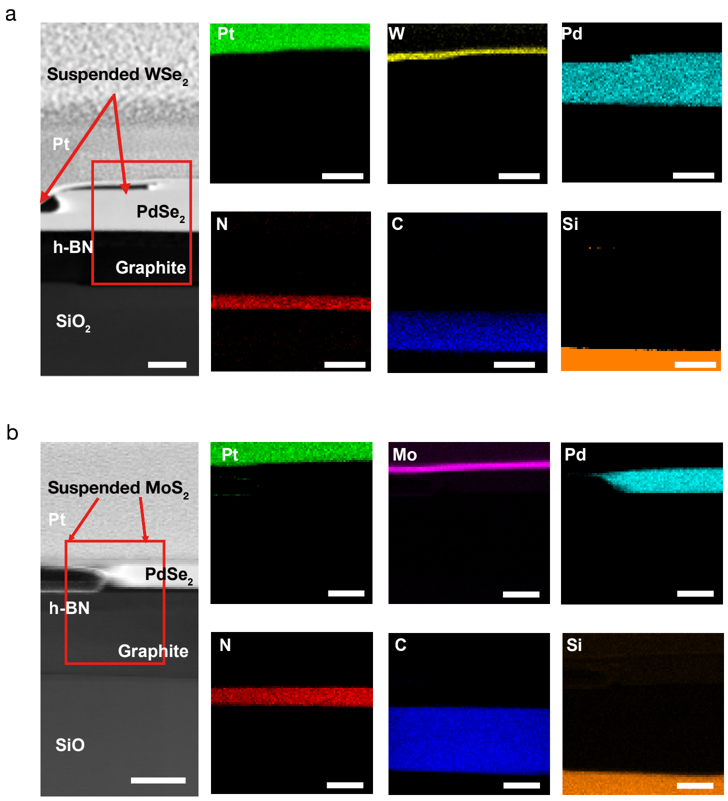

Supplementary Fig. 9 Transmission electron microscopy (TEM) image and the corresponding energy-dispersive X-ray spectroscopy in scanning transmission electron microscopy (STEM-EDS) images of the cross-section. Scale bar: 50 nm. **(a)** Cross-section of WSe_2/$PdSe_2$ junction with the airgap. **(b)** Micrograph of the cross-section of MoS_2/$PdSe_2$ junction with the airgap.

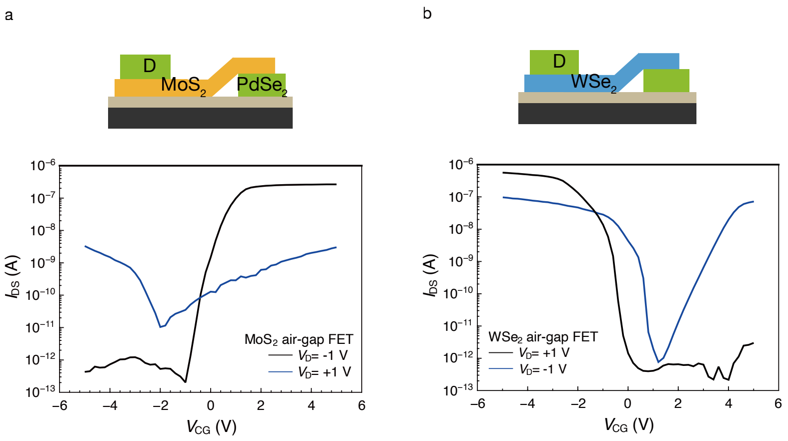

Supplementary Fig. 10 Transfer curves of offset contact devices at opposit drain voltages V_D. The top-contact $PdSe_2$ electrode served as drain electrode. **(a)** Schematic and transfer curves of MoS_2-$PdSe_2$ junction at V_D=± 1 V, transfer curve exhibites lower on-current and smaller on-off ratio at V_D = +1 V. **(b)** Schematic and transfer curves of WSe_2-$PdSe_2$ junction at V_D=± 1 V, transfer curve exhibites lower on-current and smaller on-off ratio at V_D = -1 V.

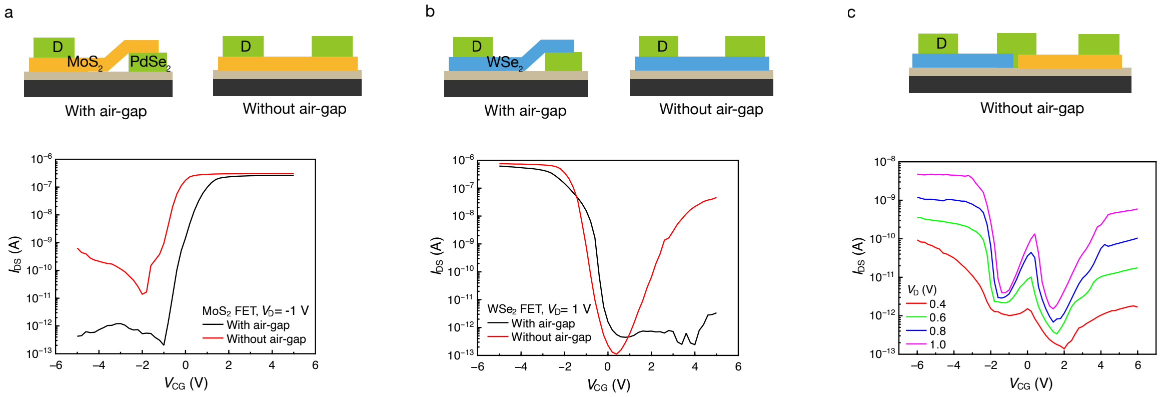

Supplementary Fig. 11 (a) The schematics and transfer curves of $PdSe_2$-MoS_2 FET with and without air-gap. **(b)** The schematics and transfer curves of $PdSe_2$-WSe_2 FET with and without air-gap. **(c)** The schematics and transfer curves of top contacted MoS_2-$PdSe_2$ and $PdSe_2$-WSe_2 in series.

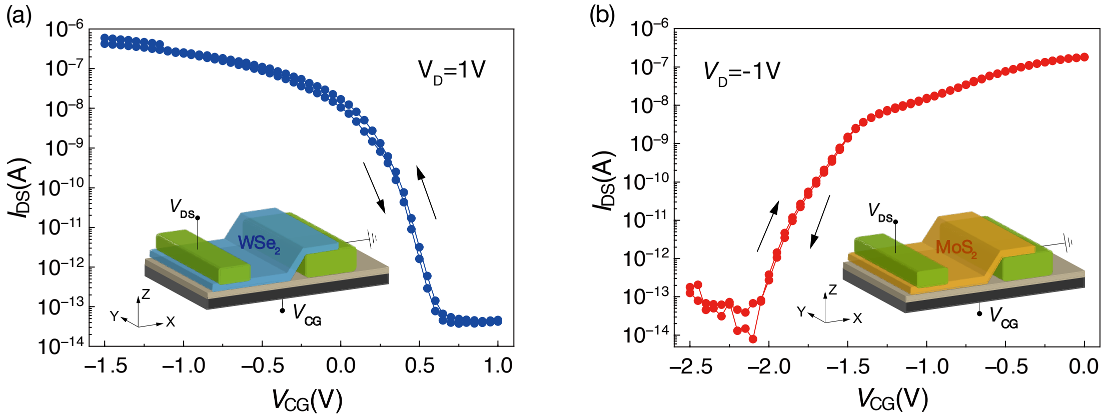

Supplementary Fig. 12 I_{DS}-V_{CG} curves of transistors with dual V_{CG} sweeps, showing a symmetric and hysteresis free transfer characteristic. **(a)** WSe_2 FET, **(b)** MoS_2 FET.

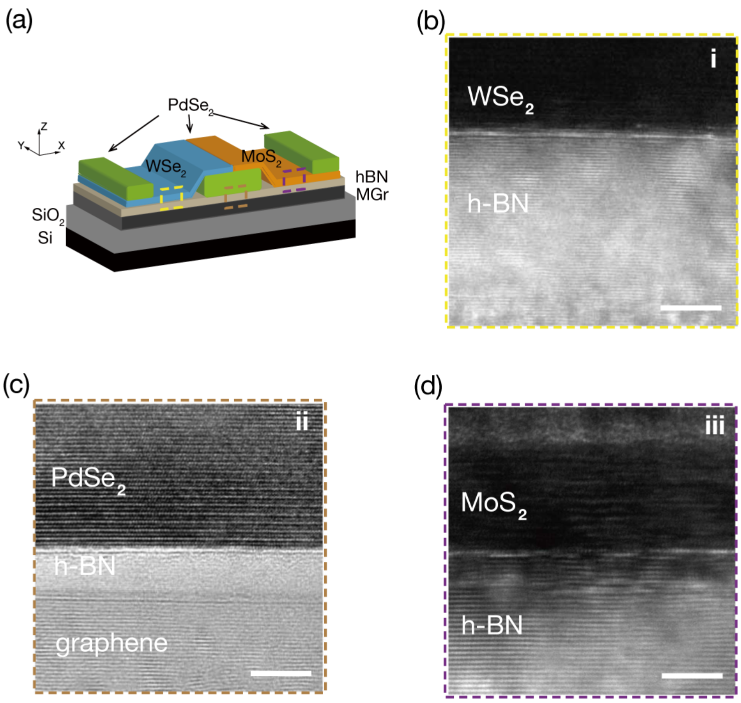

Supplementary Fig. 13 (a) Schematic of the WSe_2-$PdSe_2$-MoS_2 junction transistor. The $PdSe_2$ was alternately arranged between WSe_2 and MoS_2. **(b-d)** Cross-sectional high-resolution transmission electron microscopy (HRTEM) image of the junction with the ultra-clean interfaces. **(b)** WSe_2-BN interface, **(c)** $PdSe_2$-BN-graphene interfaces, **(d)** MoS_2-BN interface. Scale bar: 5nm.

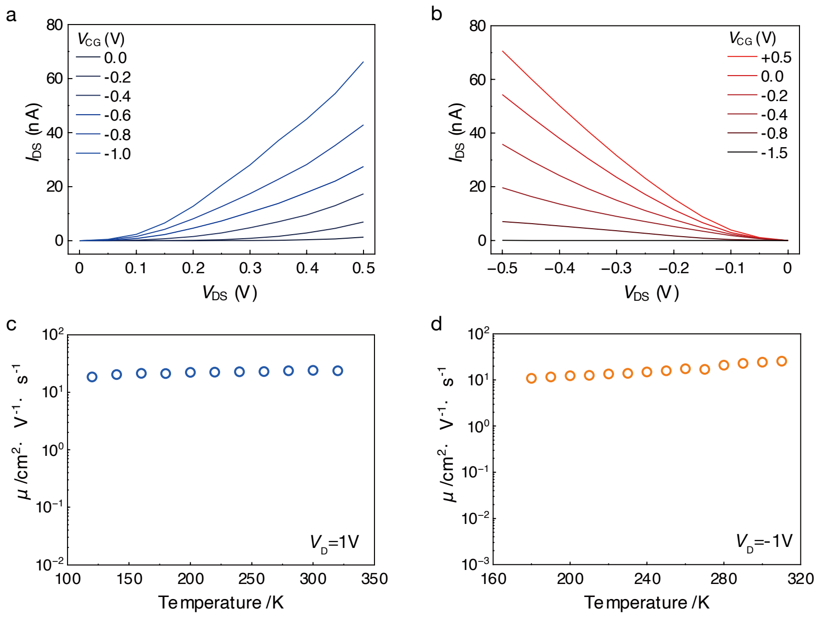

Supplementary Fig. 14 (a, b) I_{DS}-V_{DS} curves of transistors at various bias of V_{CG}. **(a)** WSe_2-$PdSe_2$ FET, **(b)** MoS_2-$PdSe_2$ FET. **(c)** Hole mobility of WSe_2 extracted from the linear region of I_{DS}-V_{CG} curves. **(d)** Electron mobility of MoS_2 extracted from the linear region of I_{DS}-V_{CG} curves.

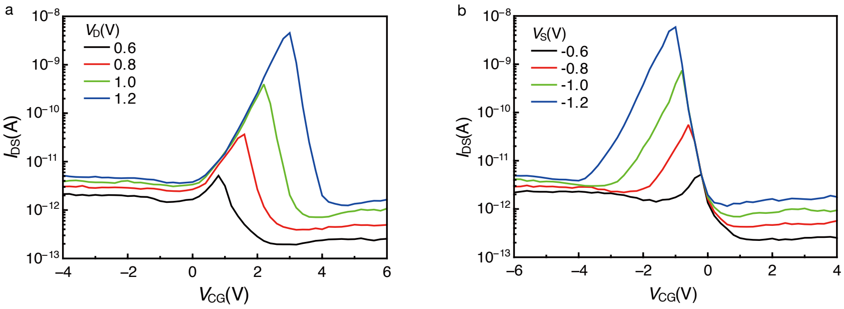

Supplementary Fig. 15 (a) The AAT curves under varying positive drain voltage (V_D) from 0.6 ~ 1.2 V. **(b)** The AAT curves under varying negative source voltage (V_S) from -0.6 ~ -1.2 V.

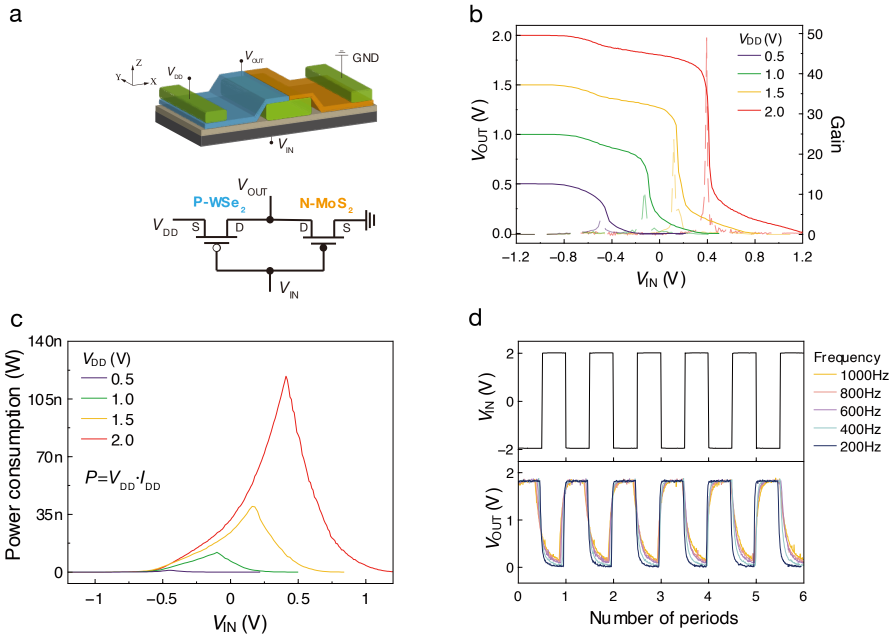

Supplementary Fig. 16 Static operation performance and dynamic switching behavior of junction inverter. **(a)** Schematic and circuit diagrams of the field-effect transistors in inverter mode. **(b)** The voltage transfer characteristics (VTCs) and voltage gain (-dV_{OUT}/ dV_{IN}) plots of the inverter at various V_{DD}. At V_{DD} = 2V, a maximum voltage gain of 49 was obtained. **(c)** The power consumption (V_{DD}×I_{DD}) of the inverter at various V_{DD}. The ultralow peak power consumption of ~1 nW was achieved at V_{DD} of 0.5 V. **(d)** The time-dependent V_{OUT} of V_{DD}=2 V driven by square wave V_{IN} with various frequencies. Logic switching behavior remained clear at a critical logic switching of 1kHz.

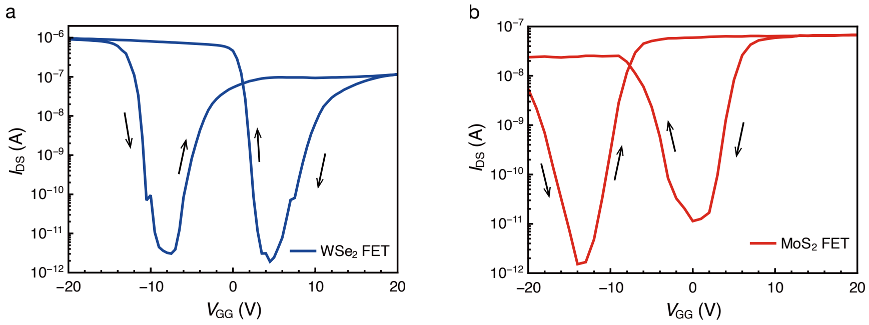

Supplementary Fig. 17 Ambipolar memory window of top contacted **(a)** WSe_2-$PdSe_2$ FGT, **(b)** MoS_2-$PdSe_2$ FGT.

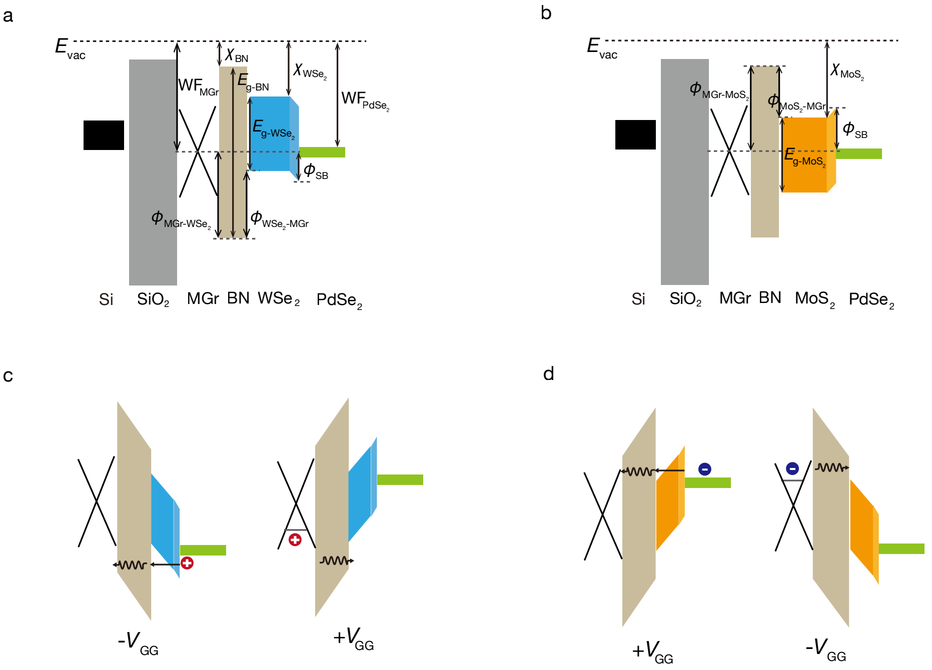

Supplementary Fig. 18 (a, b) The flat band state of the heterostructure along the injection path in **(a)** $PdSe_2$-WSe_2 floating gate, **(b)** $PdSe_2$-MoS_2 floating gate, where the χ is the electron affinity, WF is the work function, E_g is the band gap, ϕ is the tunneling barrier, and ϕ_{SB} is the Schottky barrier. **(c, d)** The programming and erasing operations to **(c)** WSe_2, **(d)** MoS_2 floating memory.

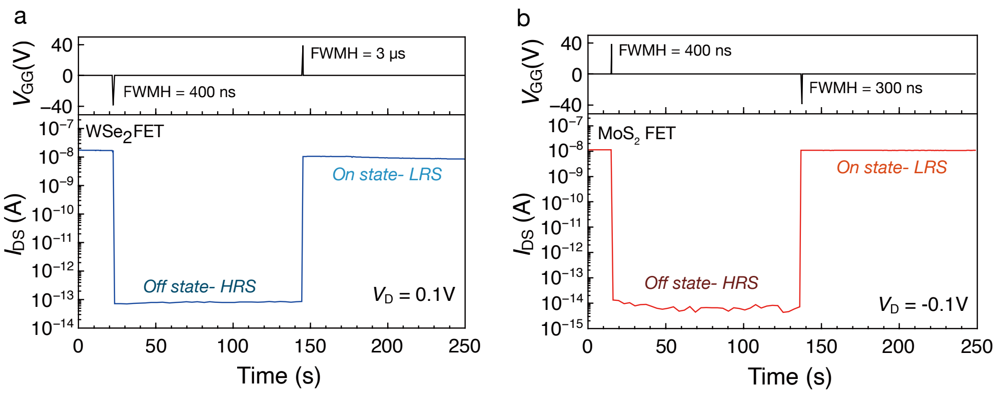

Supplementary Fig. 19 Ultrafast the program and erase performance of complementary memory when a pulse amplitude of 38 V applied to V_{GG}. **(a)** 400 ns programming, 3 μs erasing for WSe_2 floating gate memory. **(b)** 400 ns programming, 300 ns erasing for MoS_2 floating gate memory.

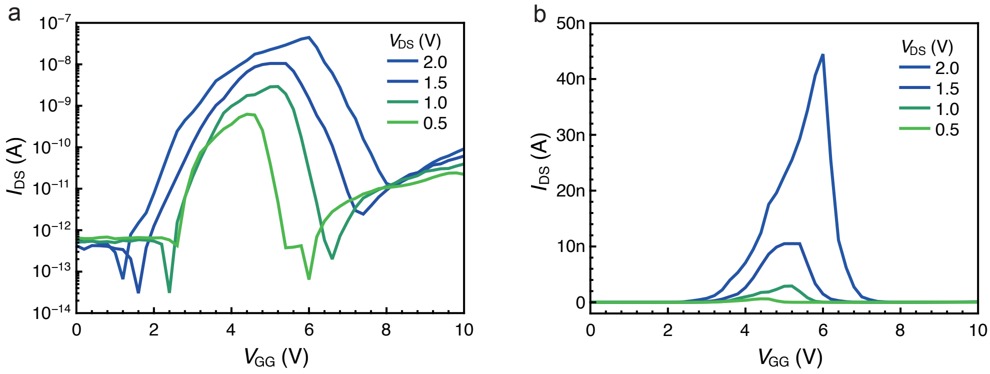

Supplementary Fig. 20 The I_{DS}-V_{GG} curves under different V_{DS}. **(a)** log scale, **(b)** linear scale.

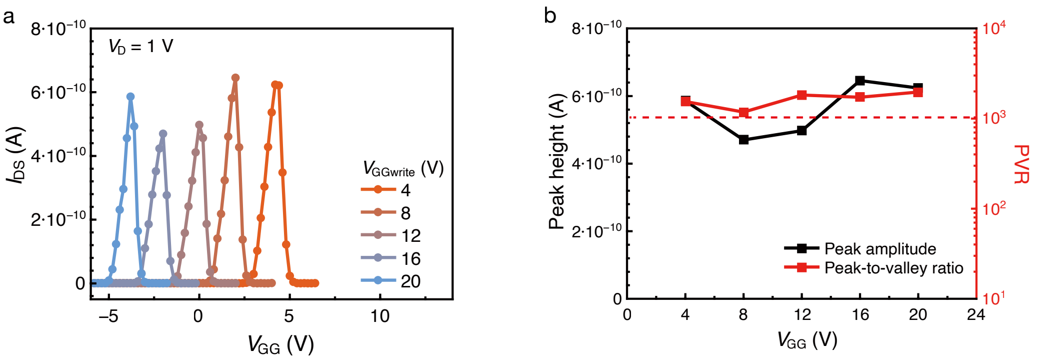

Supplementary Fig. 21 (a) Peak shifting after V_{GG} spikes with increasing amplitudes were applied in linear scale. **(b)** The peak height and peak-to-valley ratio (PVR) according to the peak shift in **(a)**.

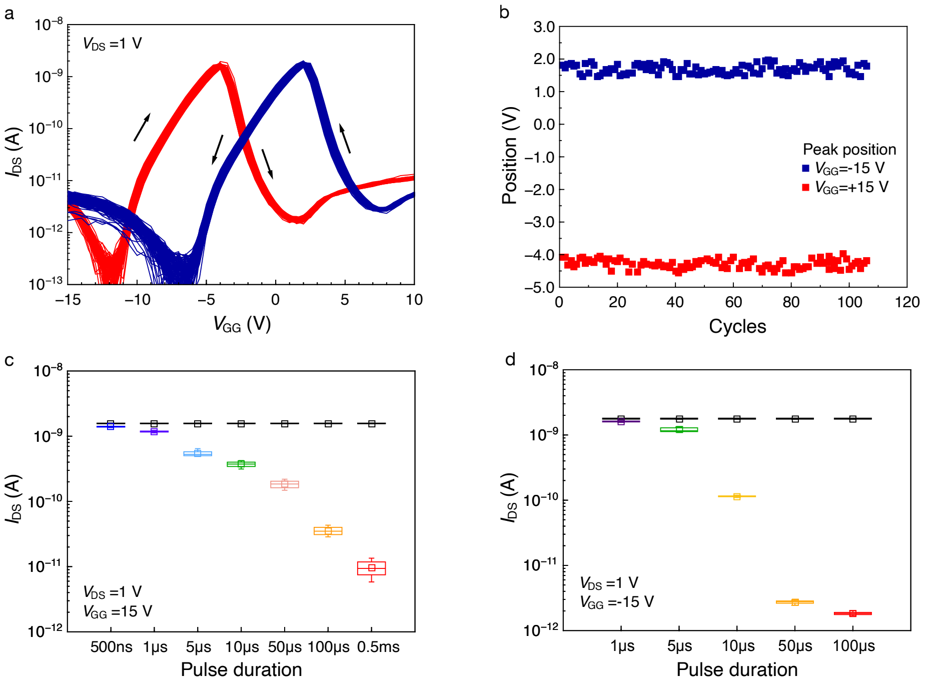

Supplementary Fig. 22 Memory performances of AATFET. **(a)** AAT curves during 110 cyclic V_{GG} round sweep from -15 V to +15 V and back to -15 V. **(b)** the writing and erasing peaks position according to the 110 endurance cycles. **(c, d)** Set peak state as initial state, the output current after applying varying duration of $V_{GG=}$ +15 V programming **(c)** and $V_{GG=}$ -15 V erasing **(d)** pulses.

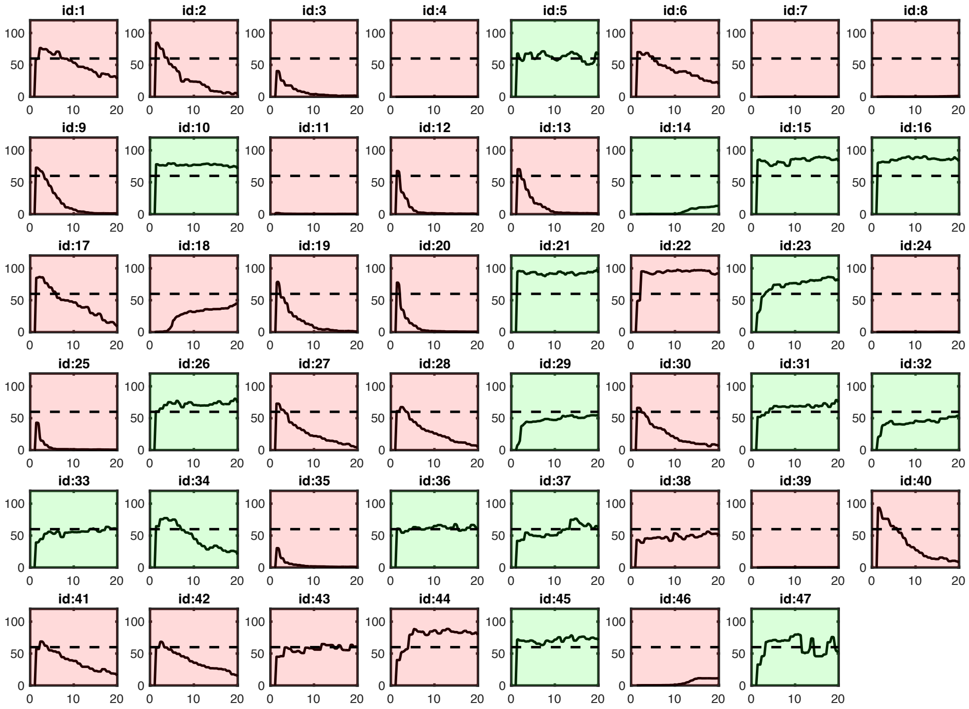

Supplementary Fig. 23 Individual device responses under the subject-specific encoded pulse as input in MIMIC-I dataset. Dash line: the threshold line training from the MIMIC-I dataset. Red color represents the abnormal cardiovascular system; Green color represents the normal cardiovascular system.

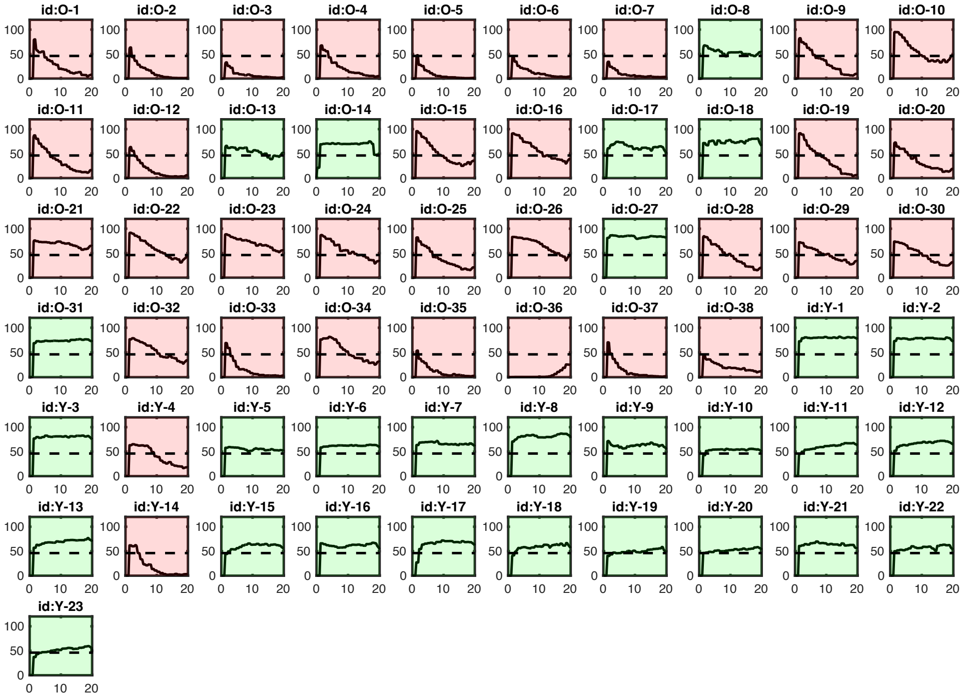

Supplementary Fig. 24 Individual device responses under the subject-specific encoded pulse as input in PWH-Elderly(O) and PWH-Young(Y) datasets. Y: young group; O: older group or elderly group in the experiments. Dash line: the threshold line training from the MIMIC-I dataset. Red color represents the abnormal cardiovascular system; Green color represents the normal cardiovascular system.

Supplementary table. 1 Energy consumption per operation

Materials	Speed	Energy consumption per operation
This work	400ns/ 300ns	19.1fJ/ 14.3fJ
MoS_2/BN/MGr[8]	4ms	771.2fJ
MoS_2/WSe_2/BN/MGr[9]	500ns	9/15 aJ
NAND Flash[10]	100μs	12nJ
MoS_2/HfO_2/Gr[11]	100ms	18pJ
MoS_2/Oxide/AlScN[12]	10μs	20pJ
MoS_2/Al_2O_3/HfO_2[13]	200ms	5.2pJ
WSe_2/$MoTe_2$[14]	1s	0.2pJ
MoS_2/HfO_2/Pt[15]	100ms	11nJ
BP/Al_2O_3[16]	100ms	40pJ
InSe/InO_x/SiO_2[17]	300ms	12pJ
WSe_2/BN/MGr[18]	1μs	0.6fJ
MoS_2/BN/MoS_2/GDYO/WSe_2[19]	20 ns	10fJ
InS_2/BN/MGr[20]	1ms	165aJ

Supplementary table. 2 Datasets summary

ID	Datasets	Subject no.	Source of measured signals	No. of normal/abmormal CVD
1	MIMIC-I [21,22]	47	A-Line continuous blood pressure waveform	17/30
2	PWH-Elderly[23]	38 (>65 yrs)	Finapres continuous blood pressure waveform	7/31
3	PWH-Young[23]	23(<35 yrs)	Finapres continuous blood pressure waveform	21/2

Supplementary References:

1. Sebastian, A., Pannone, A., Subbulakshmi Radhakrishnan, S. & Das, S. Gaussian synapses for probabilistic neural networks. *Nat. Commun.* **10**, 4199 (2019).
2. Klabunde, R. *Cardiovascular Physiology Concepts*. (Lippincott Williams & Wilkins, 2011).
3. Opie, L. H. *Heart Physiology: From Cell to Circulation*. (Lippincott Williams & Wilkins, 2004).
4. Dampney, R. A. L. *et al.* Central Mechanisms Underlying Short- And Long-Term Regulation Of The Cardiovascular System. *Clin. Exp. Pharmacol. Physiol.* **29**, 261–268 (2002).
5. Guyton, A. C. M. D. *Text Book of Medical Physiology*. (China, 2006).
6. Zill D G. Advanced engineering mathematics[M]. Jones & Bartlett Learning, 2020.
7. Dehaene S, Izard V, Spelke E, et al. Log or linear? Distinct intuitions of the number scale in Western and Amazonian indigene cultures[J]. science, 2008, 320(5880): 1217-1220.
8. Li, W. *et al.* Demonstration of Nonvolatile Storage and Synaptic Functions in All-Two-Dimensional Floating-Gate Transistors Based on MoS_2 Channels. *ACS Appl. Electron. Mater.* **5**, 4354–4362 (2023).
9. Su, Z., Cheng, H., Sun, X., Sun, H. & Zuo, C. High-Performance Floating Gate Heterostructure With WSe_2 -MoS_2 Diode Channel for Neural Synapse. *IEEE Electron Device Lett.* **44**, 1084–1087 (2023).
10. Xie, Y. Modeling, Architecture, and Applications for Emerging Memory Technologies. *IEEE Des. Test Comput.* **28**, 44–51 (2011).
11. Bertolazzi, S., Krasnozhon, D. & Kis, A. Nonvolatile Memory Cells Based on MoS_2 /Graphene Heterostructures. *ACS Nano* **7**, 3246–3252 (2013).
12. Liu, X. *et al.* Post-CMOS Compatible Aluminum Scandium Nitride/2D Channel Ferroelectric Field-Effect-Transistor Memory. *Nano Lett.* **21**, 3753–3761 (2021).
13. Zhang, E. *et al.* Tunable Charge-Trap Memory Based on Few-Layer MoS_2. *ACS Nano* **9**, 612–619 (2015).
14. Park, S. *et al.* Nonvolatile and Neuromorphic Memory Devices Using Interfacial Traps in Two-Dimensional WSe_2 /$MoTe_2$ Stack Channel. *ACS Nano* **14**, 12064–12071 (2020).
15. Migliato Marega, G. *et al.* Logic-in-memory based on an atomically thin semiconductor. *Nature* **587**, 72–77 (2020).
16. Tian, H. *et al.* A Dynamically Reconfigurable Ambipolar Black Phosphorus Memory Device. *ACS Nano* **10**, 10428–10435 (2016).

17. Yang, F.-S. *et al.* Oxidation-boosted charge trapping in ultra-sensitive van der Waals materials for artificial synaptic features. *Nat. Commun.* **11**, 2972 (2020).

18. Su, Z.-J. *et al.* Sub-femto-Joule energy consumption memory device based on van der Waals heterostructure for in-memory computing. *Chip* **1**, 100014 (2022).

19. Li, Y. *et al.* Low-voltage ultrafast nonvolatile memory via direct charge injection through a threshold resistive-switching layer. *Nat. Commun.* **13**, 4591 (2022).

20. Gao, C. *et al.* Touch-modulated van der Waals heterostructure with self-writing power switch for synaptic simulation. *Nano Energy* **91**, 106659 (2022).

21. Moody, G. B., Mark., R. G. A database to support development and evaluation of intelligent intensive care monitoring. Comput. In Cardiol., 657-660 (1996).

22. Goldberger, A. L. et al. PhysioBank, PhysioToolkit, and PhysioNet. Circulation 101, e215–e220 (2000).

23. Qiu S, Yan B P Y, Zhao N. Stroke-volume-allocation model enabling wearable sensors for vascular age and cardiovascular disease assessment[J]. npj Flexible Electronics, 2024, 8(1): 24.
